# Supplementary material for: Evidence for a Pan-Neurodegenerative Disease Response in Huntington's and Parkinson's Disease Expression Profiles
Source: Front Mol Neurosci. 2018 Jan 11;10:430. doi: 10.3389/fnmol.2017.00430 (PMC5768647; doi:10.3389/fnmol.2017.00430)
Supplement: Supplementary file 2 [file DataSheet2.PDF]

# Investigation of ENSG00000272403

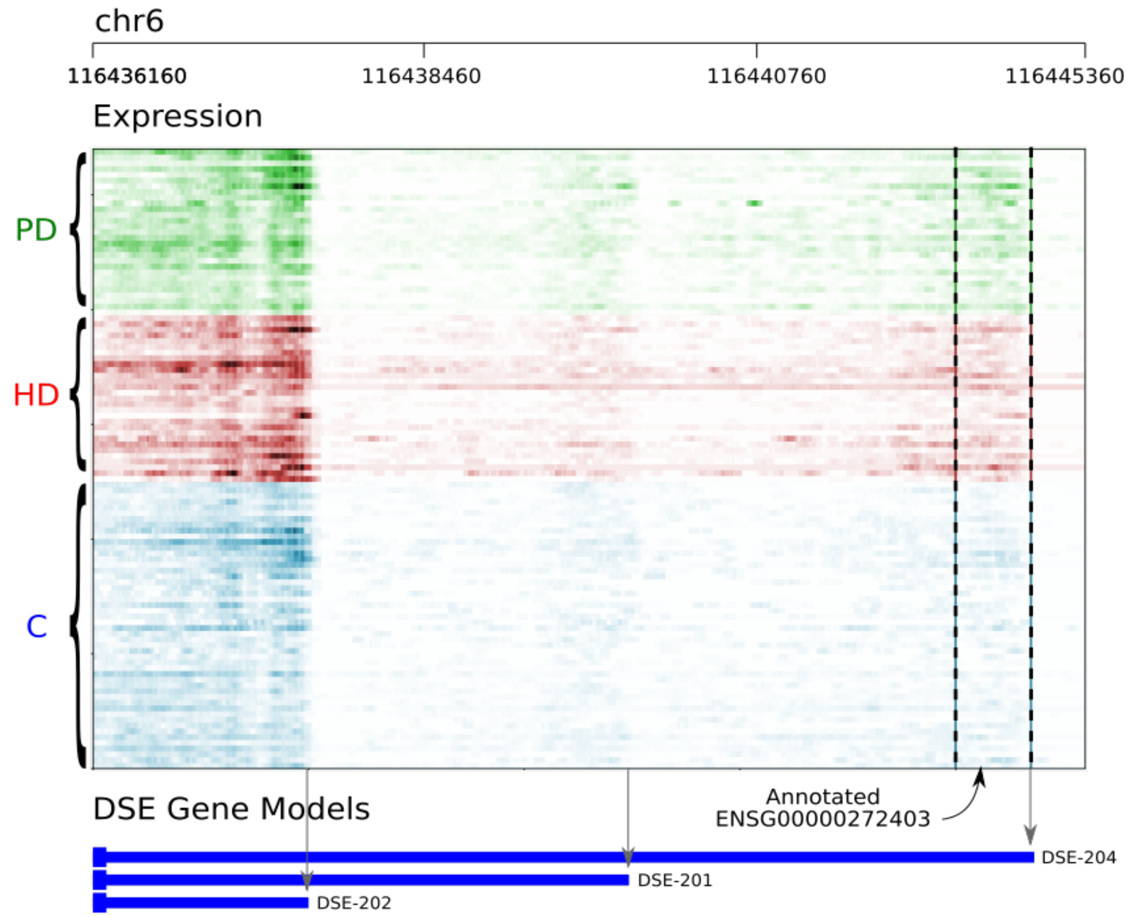


The most significant gene discovered by RRA analysis of the HD, PD, and ND gene lists was ENSG00000272403. The above figure depicts the normalized read depth for PD, HD, and C samples for the region surrounding the annotated ENSG00000272403 gene. Darker colors are proportional to increased read depth at the given genomic position. This annotation was removed from GENCODE in the release following that used in this analysis (v21). The retired annotation was for a lncRNA immediately downstream of the DSE gene. By examining the expression data with respect to the most recent annotation of DSE, we notice that the region previously annotated as ENSG00000272403 is most likely the end of the 3’ UTR for the DSE-204 transcript of DSE. The DSE gene model in GENCODE v25 has three alternative 3’ UTR termination sites, and all three isoforms end at a location where there is an observable expression signature across all samples. We can also notice an increase in expression (i.e. darker color) in the PD and HD samples compared with C at the annotated locus, consistent with the DE relationship identified by RRA. The DSE gene itself is also DE in all three gene lists. We therefore conclude that the significance of ENSG00000272403 is attributable to the same DE event as that for DSE.
